# Mortality prediction upon hospital admission – the value of clinical assessment: A retrospective, matched cohort study

**DOI:** 10.1097/MD.0000000000030917

**Published:** 2022-09-30

**Authors:** Noam Glick, Adva Vaisman, Liat Negru, Gad Segal, Eduard Itelman

**Affiliations:** a Internal Medicine “I”, Chaim Sheba Medical Center, Tel-Aviv University, Tel-Aviv, Israel.

**Keywords:** clinical evaluation, electronic medical record, EMR, mortality, prognosis

## Abstract

Accurate prediction of mortality upon hospital admission is of great value, both for the sake of patients and appropriate resources’ allocation. A myriad of assessment tools exists for this purpose. The evidence relating to the comparative value of clinical assessment versus established indexes are scarce. We analyzed the accuracy of a senior physician’s clinical assessment in a retrospective cohort of patients in a crude, general patients’ population and later on a propensity matched patients’ population. In one department of internal medicine in a tertiary hospital, of 9891 admitted patients, 973 (10%) were categorized as prone to death in a 6-months’ duration by a senior physician. The risk of death was significantly higher for these patients [73.1% vs 14.1% mortality within 180 days; hazard ratio (HR) = 7.58; confidence intervals (CI) 7.02‐8.19, *P* < .001]. After accounting for multiple, other patients’ variables associated with increased risk of mortality, the correlation remained significant (HR = 3.25; CI 2.85‐3.71, *P* < .001). We further performed a propensity matching analysis (a subgroup of 710 patients, subdivided to two groups with 355 patients each): survival rates were as low as 45% for patients categorized as prone to death compared to 78% in patients who weren’t categorized as such (*P* < .001). Reliance on clinical evaluation, done by an experienced senior physician, is an appropriate tool for mortality prediction upon hospital admission, achieving high accuracy rates.

## 1. Introduction

Accurate prediction of survival, mortality and death at admission to an internal medicine department is complex yet crucial for decision making, leading eventually to optimal patient management.^[1,2]^ It has become widespread today to incorporate life expectancy into clinical decisions, as a central factor in weighing the benefits and the burdens of diagnostic evaluations and treatment modalities^[1]^ as part of implementing personalized medicine. One may even state that prognostication plays an equal part in every clinical decision, along with diagnosis and treatment.^[3]^ Prognostication and more over identification of patients with a short life expectancy will allow for optimal treatment including a cohesive treatment plan, appropriate resources’ allocation, reassessment of care goals, focus on symptoms control, caregiver support, palliative care when relevant and hospice referral.^[4]^

In Israel, the need for mortality prediction is even legislated in a state law ‐ “The Law of the Terminally Ill”. The law states that the medical treatment, medical condition and the degree of suffering of a patient are the leading considerations in the decision-making process when deciding on medical management.^[5]^ The law settles the conflict in treating the terminally ill through balancing the value of life, the autonomy of a person and the importance of quality of life.^[5]^ The law is based on the fundamental values of the State of Israel in the fields of morality, ethics and religion.^[5]^ A patient will be categorized as terminally ill if suffering from an incurable medical condition that even with the proper medical care his or her life expectancy will not exceed six months.^[5]^ The healthcare professional eligible to determine that a certain patient is “terminally ill patient” is a physician, head of a department or head of a medical unit in the facility in which the patient is hospitalizaed.^[5]^ In compliance with this law, the electronic medical records in our hospital are equipped with a designated component termed “The Terminally Ill Patient”, which should be filled out for each patient upon admission.

It then raises the question – how can one accurately and objectively predict the survival of a hospitalized patient and how to categorize a patient as terminally ill when relevant? In some cases, this could be a difficult and not a straight forward task. According to the literature, there are available prognostic indices to aid predict mortality. Nevertheless, most of these are not incorporated routinely into the daily work of internalists since they often relate to a specific patient population and specific diseases^[4,6,7]^ (i.e. Karnofsky performance scale for malignancy^[8]^). Other stratification modalities require acquiring certain laboratory data and functional status or necessitate applying lengthy formulas.^[4]^

In this study we assessed the accuracy of mortality prediction at 6-months post hospital admission to an internal medicine department, done by a senior physician – in the crude, general patients’ population and also after propensity matching analysis.

## 2. Methods

Patients’ baseline demographic and clinical data were retrieved from their computerized records. Diagnoses were based on the International Classification of Diseases, Ninth Revision [ICD-9] codes, laboratory tests, medications, physiological signals (e.g., ECGs), radiological images (e.g., echocardiograms, angiograms), and procedures’ reports. The primary outcome of the current study was all-cause mortality. Survival data was available for all subjects from the National Israeli Population Registry. Continuous variables were expressed as mean ± standard deviation if normally distributed or median with interquartile range (IQR) if skewed. Categorical variables were presented as frequency (%). Continuous data were compared with the Student’s *t*-test and categorical data were compared with the use of the χ^2^ test or Fisher exact test. The probability of death according to the study groups was graphically displayed according to the method of Kaplan–Meier, with comparison of cumulative survival across strata by the log-rank test. Univariate Cox proportional hazards regression modeling was used to determine the unadjusted hazard ratio (HR) for all-cause mortality of patients defined as prone to death. Parameters that were found to be statistically significant in the univariate model or that are known to be significant for survival of patients were then incorporated into the multivariate model. Propensity matched analysis was performed using the nearest neighbor method comparing patients defined and not defined as prone to death. Patients were matched on demographic and clinical features. All analyses were performed using R software version 3.4.4 (R Foundation for Statistical Computing, Vienna, Austria). An association was considered statistically significant for a two-sided *P* value < .05.

## 3. Results

### 3.1. Demographic and clinical characteristics

The final study population included 9891 patients, of which, 973 (10%) were categorized as prone to death in 6-months’ duration by a senior physician. In the study population, the median age in the prone to death group was higher, (78, IQR 66‐88) and were predominantly female (514, 53%). Patients categorized as prone to death had a higher incidence of dementia (13% vs 5%, *P* < .001) and solid malignancies (51% vs 13%, *P* < .001) compared to non-prone to death patients. Considering laboratory characteristics, patients categorized as prone to death had lower median GFR values 71 mL/min (IQR 40.6‐106.8) compared to non-prone to death who had median GFR of 77 mL/min (IQR 52.8‐101.3). Table 1 include demographic and clinical characteristics of both groups and consistently demonstrate that patients categorized as prone to death more often used anticoagulants and antiepileptics and less often used antihypertensives and lipid-lowering drugs. There was no statistically significant difference in both groups regarding the incidence of congestive heart failure (CHF), chronic obstructive pulmonary disease, hematologic malignancies and chronic use of diuretics.

**Table 1:** Patients’ demographics. Whole cohort.

	Overall	Not prone to death	Prone to death	*P*
n	9891	8918	973	
Age ‐ Median (IQR)	73.2 (62.9‐82.9)	72.7 (62.6‐82.4)	77.7 (66.3‐88)	<.001
Male ‐ N (%)	5213 (53)	4754 (53)	459 (47)	<.001
BMI ‐ Median (IQR)	26 (23.1‐29.7)	26.1 (23.3‐29.9)	24.2 (21‐27.3)	<.001
DM ‐ N (%)	2818 (28)	2584 (29)	234 (24)	.001
Dyslipidaemia – N (%)	1788 (18)	1671 (19)	117 (12)	<.001
IHD – N (%)	2357 (24)	2158 (24)	199 (20)	0.01
CHF ‐ N (%)	1160 (12)	1059 (12)	101 (10)	0.186
Stroke ‐ N (%)	2138 (22)	1902 (21)	236 (24)	0.039
Dementia ‐ N (%)	551 (6)	424 (5)	127 (13)	<.001
COPD ‐ N (%)	822 (8)	750 (8)	72 (7.4)	.306
Solid Malignancy ‐ N (%)	1680 (17)	1185 (13)	495 (51)	<.001
Hem. malignancy ‐ N (%)	335 (3)	307 (3)	28 (2.9)	.406
Antihypertensive ‐ N (%)	5175 (52)	4794 (54)	381 (39)	<.001
Antiaggregant ‐ N (%)	4271 (43)	3982 (45)	289 (30)	<.001
Antiepileptic ‐ N (%)	1191 (12)	1032 (12)	159 (16)	<.001
Lipid lowering ‐ N (%)	4421 (45)	4191 (47)	230 (24)	<.001
Diuretics ‐ N (%)	2451 (25)	2221 (25)	230 (24)	.407
Anti coagulant ‐ N (%)	750 (8)	640 (7)	110 (11)	<.001
HGB ‐ median (IQR)	11.93 (10.3‐13.2)	12.03 (10.5‐13.3)	10.5 (9.1‐12.1)	<0.001
eGFR ‐ median (IQR)	76.5 (51.7‐101.6)	77 (52.8‐101.3)	71.1 (40.6‐106.8)	0.005

BMI = body mass index, CHF = congestive heart failure, COPD = chronic obstructive pulmonary disease, DM = diabetes mellitus, eGFR = estimated glomerular filtration rate, HEM = hematologic, HGB = hemoglobin, IHD = ischemic heart disease, IQR = inter-quartile range.

### 3.2. Association of defining patients as prone to death with actual mortality rates

**Figure 1.**
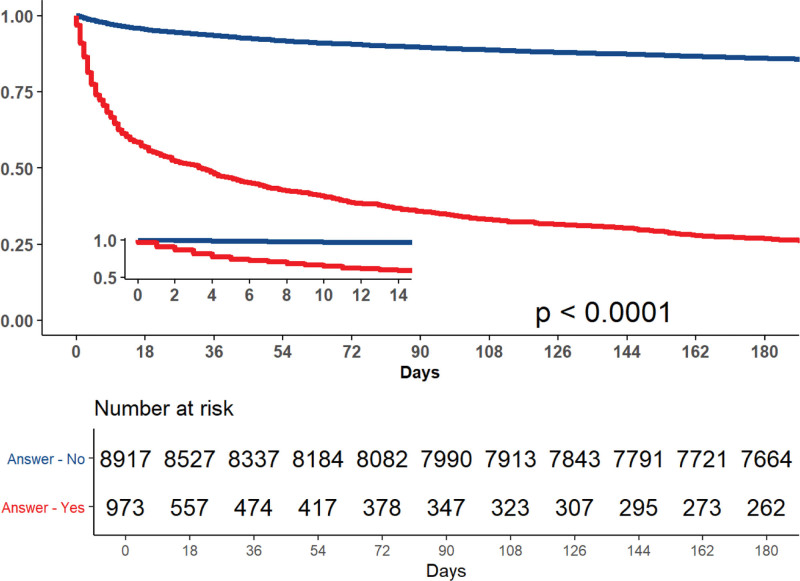
Cumulative survival. Whole cohort.

As demonstrated by the Kaplan–Meier survival analysis (Fig. 1), the cumulative probability of survival was significantly lower in patients categorized as prone to death. As shown, the probability of survival 180 days after categorization (made no longer than 48 hours following admission) was 26.9% versus 85.9% (*P* < .001) for prone to death and non-prone to death groups, respectively. As soon as 18 days after categorization, increased mortality 42.8% (390) was observed in the prone to death group, compared to only 4.4% (416) patients in the non-prone to death group (*P* < .001).

### 3.3. Univariate analysis

Consistently, univariate analysis shown in Table 2 reveals that patients categorized as prone to death were overall 7.58 times more likely to die within the follow-up period compared to patients who weren’t categorized as such (CI 7.02‐8.19, *P* < .001). Age increased the risk of death by 1.04-fold (CI 1.04‐1.05; *P* < .001) for every year of life. Comorbidities associated with increased risk of death were diabetes mellitus (HR = 1.26, CI 1.18‐1.35; *P* < .001), ischemic heart disease (IHD) (HR = 1.34, CI 1.26‐1.44; *P* < .001), solid malignancies (HR = 2.51, CI 2.34‐2.69; *P* < .001), dementia (HR = 2.77, CI 2.51‐3.06; *P* < .001) and hematologic malignancies (HR = 1.35, CI 1.16‐1.56; *P* < .001). The use of certain medications such as diuretics (HR = 1.87, CI 1.76‐2.00; *P* < .001), anticoagulants (HR = 1.64, CI 1.48‐1.81; *P* < .001) and antiepileptics (HR = 1.27, CI 1.16‐1.38; *P* < .001) were also associated with increased mortality. Factors found to have an inverse relation to the risk of death included elevated body-mass index (BMI) (HR = 0.96, CI 0.95‐0.97; *P* < .001) use of lipid-lowering drugs, increased hemoglobin levels (HR = 0.8, CI 0.79‐0.81; *P* < .001) and glomerular filtration rate (GFR). Statistically insignificant factors included patients’ gender, dyslipidemia and use of antihypertensives or anti-aggregants.

**Table 2:** Univariate analysis of patients’ characteristics.

	HR [CI]	*P*
Prone to death	7.58 [7.02, 8.19]	<.001
Age	1.04 [1.04, 1.05]	<.001
Male	0.99 [0.93, 1.05]	.796
BMI	0.96 [0.95, 0.97]	<.001
DM	1.26 [1.18, 1.35]	<.001
Dyslipidaemia	0.93 [0.86, 1.01]	.069
IHD	1.34 [1.26, 1.44]	<.001
COPD	1.60 [1.46, 1.76]	<.001
Solid malignancy	2.51 [2.34, 2.69]	<.001
Dementia	2.77 [2.51, 3.06]	<.001
Hematologic malignancy	1.35 [1.16, 1.56]	<.001
Anti hypertensive	1.04 [0.98, 1.11]	.195
Anti aggregant	0.95 [0.90, 1.01]	.133
Anti epileptic	1.27 [1.16, 1.38]	<.001
Lipid lowering	0.74 [0.69, 0.78]	<.001
Diuretics	1.87 [1.76, 2.00]	<.001
Anti coagulant	1.64 [1.48, 1.81]	<.001
HGB	0.80 [0.79, 0.81]	<.001
eGFR (MDRD)	0.99 [0.99, 0.99]	<.001

BMI = body mass index, CI = confidence interval, COPD = chronic obstructive pulmonary disease, DM = diabetes mellitus, eGFR = estimated glomerular filtration rate, HGB = hemoglobin, HR = hazard ratio, IHD = ischemic heart disease, IQR = inter-quartile range, MDRD= modification of diet in renal disease.

### 3.4. Multivariate and subgroup analysis

A multivariate model adjusted to different demographic and clinical characteristics presented in Table 3 was constructed to stratify for important predictors of mortality. The analysis demonstrates that being categorized as prone do death by a clinician, upon admission, increases one’s risk of death by 3.25-fold (CI 2.85‐3.71; *P* < .001). Risk of death was also increased in elderly and in men: for each added year of life, the risk of death in patients categorized as prone to death was increased by 1.03-fold (CI 1.02‐1.03, *P* < .001). Being a male imposed a risk of 1.17-fold (CI 1.08‐1.28, *P* < .001). Clinical background of dementia (HR = 1.47, CI 1.26‐1.71; *P* < .001), CHF (HR = 1.57, CI 1.41‐1.75; *P* < .001), Diabetes Mellitus (HR = 1.10, CI 1.01‐1.21; *P* = .031), also increases risk of death. Suffering from a solid malignancy increased the risk of death by 1.78-fold (CI 1.62‐1.96; *P* < .001) while suffering from a hematologic malignancy increases risk of death by 1.29-fold (CI 1.08‐1.55; *P* = .006). Regarding laboratory parameters, hemoglobin level was inversely related to risk of death: for every 1 g/dL added, the risk of death was decreased by 0.88-fold (CI 0.87‐0.9, *P* < .001). The risk of death was rising with lower GFR values (<30 mL/min, HR = 1.43, CI 1.27‐1.62; *P* < .001). Past history of stroke and IHD was statistically insignificant for the risk of consequent death (*P* = .714 and *P* = .506 respectively).

**Table 3:** Multivariate analysis of patients’ characteristics.

	HR [CI]	*P*
Prone to death	3.25 [2.85, 3.71]	<.001
Age	1.03 [1.02, 1.03]	<.001
Male	1.17 [1.08, 1.28]	<.001
BMI	0.97 [0.96, 0.98]	<.001
DM	1.10 [1.01, 1.21]	.031
Dyslipidaemia	0.89 [0.80, 0.99]	.026
IHD	0.97 [0.88, 1.07]	.506
CHF	1.57 [1.41, 1.75]	<.001
Stroke	0.98 [0.89, 1.08]	.714
Dementia	1.47 [1.26, 1.71]	<.001
COPD	1.41 [1.24, 1.60]	<.001
Solid malignancy	1.78 [1.62, 1.96]	<.001
Hematologic malignancy	1.29 [1.08, 1.55]	.006
HGB	0.88 [0.87, 0.90]	<.001
eGFR (MDRD) < 30	1.43 [1.27, 1.62]	<.001

BMI = body mass index, CI = confidence interval, COPD = chronic obstructive pulmonary disease, DM = diabetes mellitus, eGFR = estimated glomerular filtration rate, HGB = hemoglobin, HR = hazard ratio, IHD = ischemic heart disease, IQR = inter-quartile range, MDRD= modification of diet in renal disease.

### 3.5. Propensity score matching

**Figure 2.**
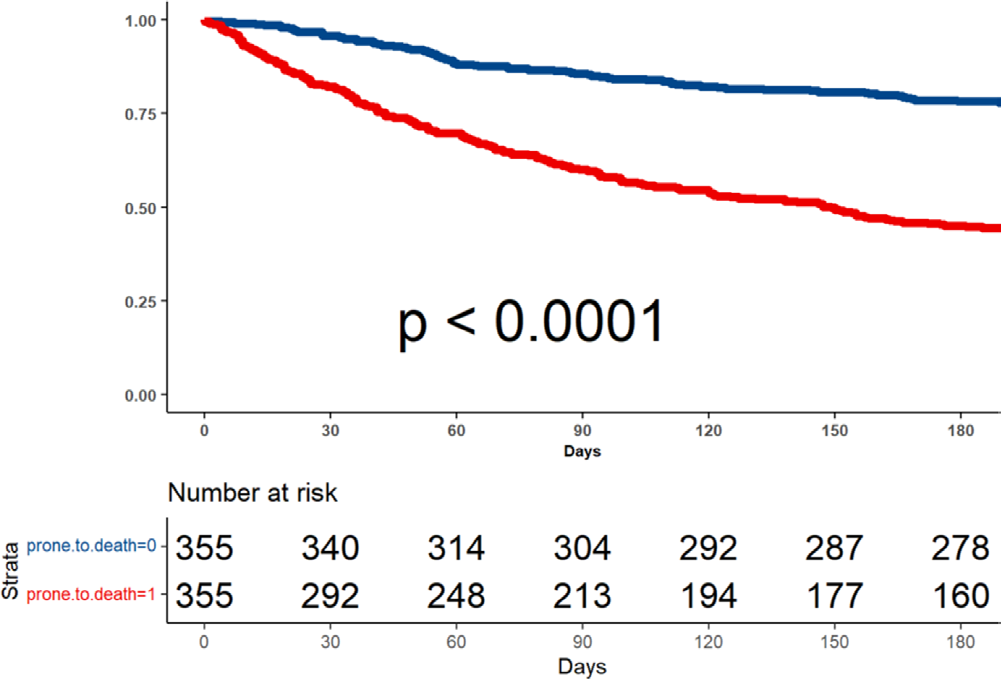
Cumulative survival. Matched population.

The study population was matched using a 1:1 nearest neighbor technique. A total of 355 patients categorized as prone to death with known comprehensive demographic, clinical, and laboratory data, were identified and matched with 355 control patients. Thus, the final matched cohort population included 710 patients. The quality of matching is presented graphically in Figure S1, Supplementary Digital Content http://links.lww.com/MD/H447. The survival in the matched population is presented in Figure 2 and demonstrates that 180 days after categorization the survival rates were as low as 45% in patients categorized as prone to death compared to survival rates of 78% in patients who weren’t categorized as such (*P* < .001). As soon as 30-days after categorization, there was already a visible, statistically significant difference of mortality between the two matched-patient groups (17.75% vs 4.23%, HR = 3.39; *P* < .001). Univariate analysis of the matched cohort populations showed that a patient categorized as prone to death had a 2.86-fold increased risk of death (CI 2.38‐3.43; *P* < .001).

## 4. Discussion

Despite the fact that early prognostication of hospitalized patients in general, internal-medicine departments is of utmost importance, the literature is inconclusive. Some authors claim that physicians can only moderately predict patient outcomes^[9–12]^ while others provide oppositional data.^[13–16]^ Our study provides supporting data of the latter practice. Hence, the professional experience of a senior physician can make an individual assessment of each patient and provide a more wholesome and accurate picture of the patient when mortality is the outcome.

In our study population, in a univariate model, prior to matching, patients prone to death were typically older, mostly female, suffered from comorbidities such as dementia, stroke, solid malignancies and more often used anti-epileptics and anticoagulants. These patients had lower hemoglobin levels and worse renal function. Patients with lower BMI were also more likely to be at higher risk for death, serving as a marker for sarcopenia and frailty. These results correlated with pervious prediction studies.^[2,17,18]^ A patient was more likely to be prone to death if there were neurological deficits upon admission, whereas cardiovascular disease such IHD of CHF resulted in a higher likelihood of survival. Still, the factor associated with death in the most statistically significant manner was a senior physician’s assessment. The risk of death was increased in patients categorized as prone to death after propensity matching and even when accounting for multiple variables.

According to our results, as early as 18 days after categorizing a patient as prone to death by a senior physician, the risk of death was already, significantly increased compared to patients who weren’t categorized as such. These two survival curves only continued to separate during the following 180 days.

We claim that the professional experience of a senior physician can provide a wholesome and accurate picture of the patient when mortality is the outcome. These results can perhaps be explained by the ability of an experienced physician to asses a patient as a whole, account physical variables as well as mental and socioeconomical ones, observe the patient’s response to immediate interventions such as medical and therapeutic procedures. The physician intuition, upon “eyeballing the patient”, based on experience built in witnessing patients is a variable that cannot be calculated and integrated in fixed models. Physical aspects such as skin paleness, breathing patterns and level of consciousness and anxiety are easily overlooked when evaluated by models based on pure physiological parameters. Environmental factors such as the patient’s support system and socioeconomic status are other aspects that cannot be straightforward calculated and integrated in a prediction score.

Altogether, the ability of a physician to “eyeball” a patient perhaps is the biggest advantage over a calculated model, as the whole is greater than the sum of its parts.

## 5. Conclusions

Out study results show that the evaluation of a senior physician is significantly accurate at predicting 6-month mortality and should be incorporated into the wholistic patient evaluation upon hospital admission.

## 6. Study limitations

This study was a retrospective observational one. Another limitation is that it was conducted in only one medical center and compared the evaluation of only one expert physician.

## Authors contributions

**Conceptualization:** Noam Glick, Adva Vaisman, Liat Negru, Gad Segal.

**Data curation:** Noam Glick, Adva Vaisman, Eduard Itelman.

**Formal analysis:** Noam Glick, Adva Vaisman, Gad Segal, Eduard Itelman.

**Investigation:** Noam Glick, Adva Vaisman, Liat Negru.

**Methodology:** Noam Glick, Adva Vaisman, Gad Segal, Eduard Itelman.

**Project administration:** Noam Glick, Adva Vaisman, Gad Segal.

**Supervision:** Noam Glick, Adva Vaisman, Gad Segal.

**Software:** Gad Segal, Eduard Itelman.

**Resources:** Adva Vaisman, Gad Segal.

**Validation:** Noam Glick, Eduard Itelman.

**Funding acquisition:** Gad Segal.

**Writing – original draft:** Noam Glick, Adva Vaisman, Liat Negru, Gad Segal, Eduard Itelman.

**Writing – review &amp; editing:** Noam Glick, Adva Vaisman, Liat Negru, Gad Segal, Eduard Itelman.

## Supplementary Material


