# Supplementary figures and images for: Mortality prediction upon hospital admission – the value of clinical assessment: A retrospective, matched cohort study
Source: Medicine (Baltimore). 2022 Sep 30;101(39):e30917. doi: 10.1097/MD.0000000000030917 (PMC9524893; doi:10.1097/MD.0000000000030917)

**Supplementary figure 1.** Quality of machining

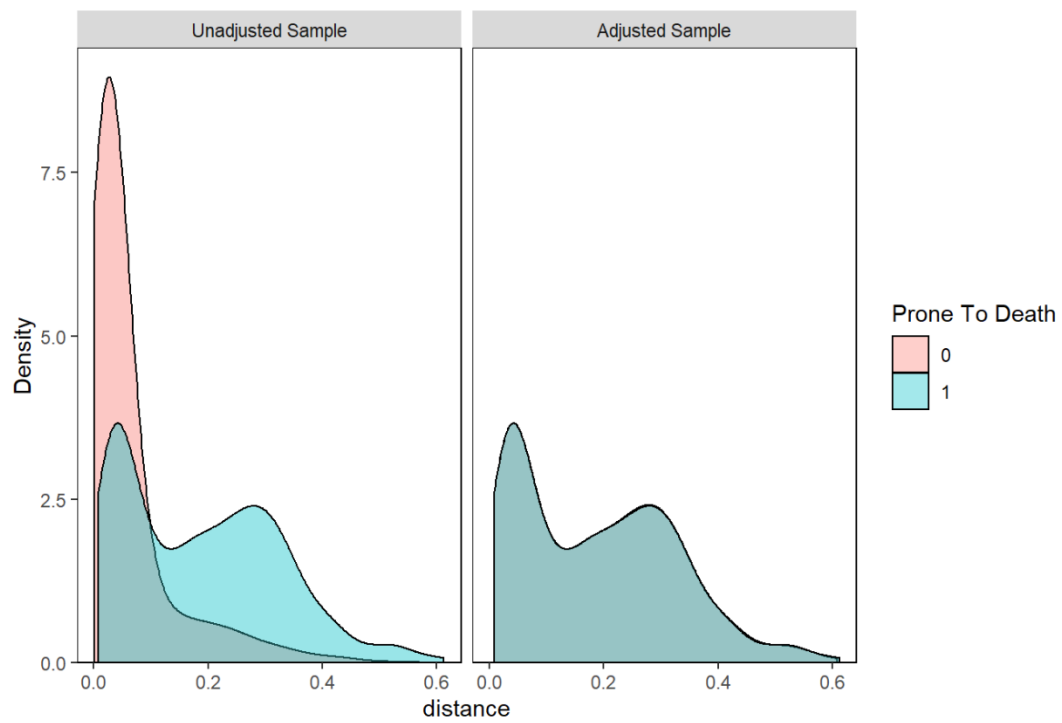

Supplement: Supplementary file 1 [file medi-101-e30917-s001.pdf]
